# Influence of PCOS in Obese vs. Non-Obese women from Mesenchymal Progenitors Stem Cells and Other Endometrial Cells: An in silico biomarker discovery

**DOI:** 10.6026/97320630013111

**Published:** 2017-04-30

**Authors:** Ashvini Desai, Inamul Hasan Madar, Amjad Hussain Asangani, Hussain Al Ssadh, Iftikhar Aslam Tayubi

**Affiliations:** 1 Department of Bioinformatics, School of Biosciences and Technology, Vellore Institute of Technology, Vellore, 632014, Tamil Nadu, India;; 2 Department of Biotechnology & Genetic Engineering and Department of Biochemistry, Bharathidasan University, Tiruchirappalli, 620024, Tamil Nadu, India;; 3 Department of Biochemistry, Islamiah College, Vaniyambadi 635 752, Vellore Dist, Tamil Nadu India;; 4 School of Biological sciences, University of Essex, Colchester, CO43SQ, United Kingdom;; 5 Faculty of Computing and Information Technology, King Abdul-Aziz University, Rabigh-21911, Saudi Arabia.

**Keywords:** Polycystic ovary syndrome (PCOS), Obesity, Differential expressed gene

## Abstract

Polycystic ovary syndrome (PCOS) is endocrine system disease which affect women ages 18 to 44 where the women’s hormones are imbalance. Recently it has been reported to occur in early age. Alteration of normal gene expression in PCOS has shown negative effects on long-term health issues. PCOS has been the responsible factor for the infertility in women of reproductive age group. Early diagnosis and treatment can improve the women’s health suffering from PCOS. Earlier Studies shows correlation of PCOS upon insulin resistance with significant outcome, Current study shows the linkage between PCOS with obesity and non-obese patients. Gene expression datasets has been downloaded from GEO (control and PCOS affected patients). Normalization of the datasets were performed using R based on RMA and differentially expressed gene (DEG) were selected on the basis of p-value 0.05 followed by functional annotation of selected gene using Enrich R and DAVID. The DEGs were significantly related to PCOS with obesity and other risk factors involved in disease. The Gene Enrichment Analysis suggests alteration of genes and associated pathway in case of obesity. Current study provides a productive groundwork for specific biomarkers identification for the accurate diagnosis and efficient target for the treatment of PCOS.

## Background

Polycystic ovary syndrome (PCOS) is one of the most common metabolic, hormonal and reproductive disorders among women of the reproductive age. Women suffering from PCOS are subjected to a range of symptoms associated with menstrual dysfunction, excess of androgen, which significantly influence the quality of life. Earlier studies reveal PCOS affects between 5% and 10% of women ages 18 to 44 [1] and the criteria used to define PCOS are frequently in change, the projected figure of affected women to be about one in every 10 to 15 women. Most women are diagnosed during their twenties or thirties, but recent studies warns that PCOS may affect even prior to age of teens and as young as 11 years of age, much ahead of their puberty [2].

The morbidities of PCOS are also linked to various associated disease or disorders, which is not limited to obesity, insulin resistance, Type II Diabetes Mellitus, cardiovascular diseases, infertility, cancer and psychological wellbeing [3]. One such longterm morbidity was studied with reference to obesity. Women who are more likely to early on-set of PCOS are due to gene inherited with PCOS parent and obesity influence to on-set of the disease. The symptoms are characterized by anovulation, hyperandrogenism and polycystic-appearing ovaries, are the most common endocrine disorder among reproductive-age women and are a leading cause of female infertility [4]. Although anovulation and impaired oocyte maturation are the main causes of decreased fecundity in PCOS, several abnormalities in PCOS endometrium have been reported, including aberrant steroid hormone action with high sex steroid receptor and coactivator expression, low expression of αvβ3 integrin, abnormal immune cell trafficking, and resistance to progesterone (P4) [5,6]. These changes likely contribute to reduce endometrial receptivity, subfertility, and poor pregnancy outcome in women with PCOS [7].

Endometrial cells respond to ovarian-derived steroid hormones with follicular phase estradiol (E2) driving endometrial cellular proliferation that is curtailed by corpus luteumP4 production in the secretory phase. In an ovulatory disorders such as in PCOS, an E2-dominant environment prevails because ovulation and P4 production are infrequent or completely absent. This results in the increased risks of endometrial hyperplasia and endometrial cancer in PCOS women [8,9]. It can be postulated that an endometrial disease phenotype is promoted by anovulation and aggravated by hyperandrogenism and metabolic and inflammatory changes related to obesity, insulin resistance, and accompanying hyperinsulinemia, all common in PCOS women [10].

Endometrial mesenchymal stem cells (eMSCs), presumptive progenitors of eSFs, reside in the perivascular space in human endometrium, and likely contribute to endometrial cyclic regeneration and lineage-specific differentiation [11,12]. Adult stem cells exist in a niche that maintains their stemness or signals their differentiation can be affected by changes in their microenvironment that may lead to abnormal lineage progeny [13]. Because inflammation and metabolic and endocrine abnormalities prevail in women with PCOS, the purpose of the current study was to determine whether the gene expression profile of specific endometrial cell populations, including eMSCs, in PCOS endometrium could give insights into the origin of endometrial abnormalities and sub fertility common in PCOS women.

Bioinformatics provides different platforms to analyses the data using in silico approach to predict differential expression level of genes in various condition. Microarray allows studying the genes of an organism under different conditions in a single experiment. The analysis of such data is performed using R, which as a matter of fact has become the standard in the field of biomarker discovery [14].

A major part of women suffering from PCOS can be reduced by early diagnosis and effective treatment methods. There is no specific test to definitely diagnose PCOS; however physician performs certain medical tests, examination like physical exam, Pelvic exam, blood tests and ultrasound imaging tests. Apart from the mentioned conventional diagnostic method, biomarkers may serve as confirmative diagnostic method for PCOS and further add to the development of the new molecular targets for drug development.

Earlier research has successfully identified genes related to PCOS and other morbidities like hyperandrogenemia and especially insulin resistance. However, a detailed study on PCOS with obesity as the major symptom is not often considered. In order to identify biomarkers for PCOS caused due to obesity specifically, dataset related to obesity with PCOS was selected for the current study. The first objective of the study is to add genes to the currently existing DEGs list. The second objective is to find out the relation between obesity associated with PCOS. To achieve the above objectives gene wide analysis of the microarray gene expression profiling was analyzed.

## Methodology

### Data and Data source

All the gene expression datasets were downloaded from NCBI’s GEO database (http://www.ncbi.nlm.nih.gov/sites/GDSbrowser). The dataset GSE48301 was downloaded which consists of two types of samples taken from obese control patients and obese PCOS patients. Total 29 samples out of which 14 samples were PCOS and 15 samples were control respectively. The datasets were downloaded in .CEL format and were analyzed on R (3.2.1). Most of the functionality in R is in the extension packages. Most of the MA analysis packages can be found on Bioconductor (https://www.bioconductor.org/) Bioconductor has been largest growing platform for the analysis and comprehension of highthroughput genomic data. R statistical programming language supports most of all the Bioconductor packages, and is open source and open development.

### Data Normalization

Normalization includes preprocessing procedure, which aims to help for any systematic differences between genes or arrays. In this work, the data normalization was performed using RMA [15]. Typically preprocessing methods, such as RMA, consist of several steps: background correction, normalization of probes, and summarization. Where individual probes are combined into a probe set. RMA is useful for highly precise estimates of expression.

### Differentially expressed genes (DEGs) extraction

The differentially expressed genes extraction result is achieved using the DEGs extraction process. The extraction of gene on DEG is based on the criteria limited to p-value 0.05 which was perform based on t-test on the normalized resultant data. The volcano plot arranges genes along dimensions of biological and statistical significance.

### Gene Annotation:

Online gene annotation tool such as EnrichR was used for differentially expressed genes annotation in order to correlate their relation with biological pathways of diseases in which they are enriched. The results were analyzed with the previous studies and knowledge based on acquired graphical results from EnrichR. Futuremore, The Database for Annotation, Visualization and Integrated Discovery (DAVID) online tool was used further for gene enrichment and extract the enriched gene names. The count threshold (minimum count) of 2 was used to retrieve minimum gene counts belonging to a GO term. An EASE Score threshold (maximum probability)/p-value <= 0.1 was used for the strong gene set enrichment result. For obtaining optimal results the count threshold (minimum count) of 2 was used to retrieve minimum gene counts belonging to a GO term. An EASE Score threshold (maximum probability)/p-value <= 0.1 was used for the optimal gene set enrichment.

## Results

**Figure 1:**
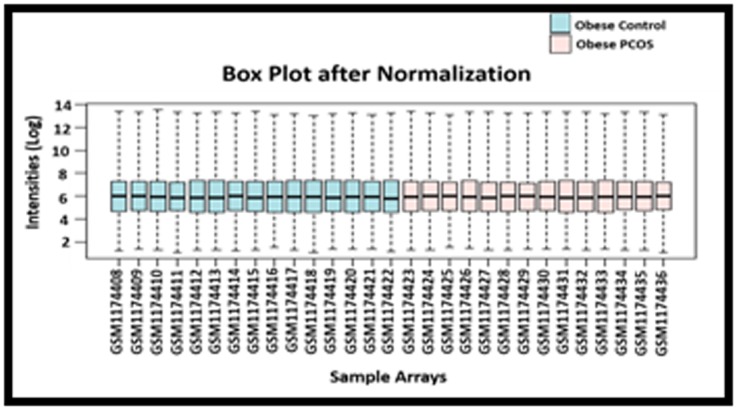
The boxplot showing normalized intensities (Log) and the distribution of 14 obese women with PCOS & 15 obese women without PCOS samples arrays.

The data was subjected to systematic biases and normalization, which was performed using RMA method. The box plot obtained in this step shows the normalized hybridization intensities across all the samples in the dataset (Figure 1).

There are 28 differentially expressed genes namely KIAA0040, CCDC3, CXCL12, SLITRK6, ANKFN1, ATP8B1, LRRTM4, ROBO2, EPHA6, GPAT3, PDLIM5, HHIP, EPHA5, NDNF, SLC27A6, FGF10, PLA2G7, ADGRF5, AHR, HGF, WNT2, NRG1, ANGPT2, FREM1, OGN, PAPPA-AS1, PAGE4 and GABRA3 were identified between both the groups, i.e. obese women without PCOS and obese women with PCOS with the adjusted pvalue of 0.05 and FC value of 0.06.

**Figure 2:**
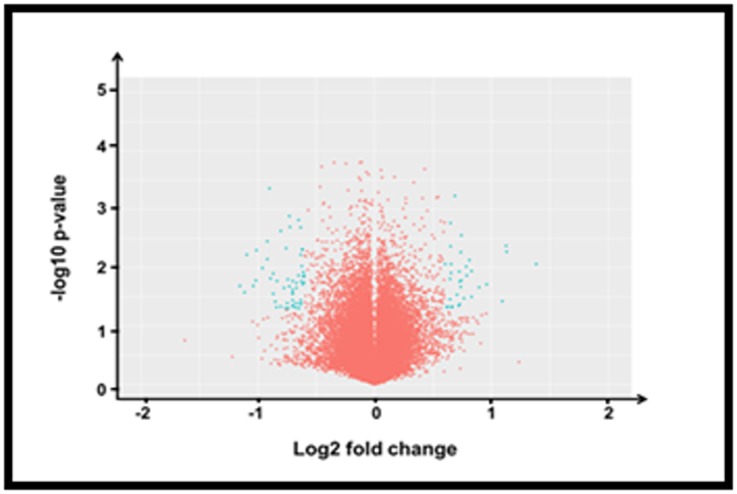
A comparison of two-gene selection method in a volcano plot. Each circle corresponds to one gene. The figure represents the average log-ratio (log fold-change) in the twogroup comparison. The 2-fold change method selects all genes above the line x=0.5 and below the line x=-0.5, as differentially expressed ones.

**Figure 3:**
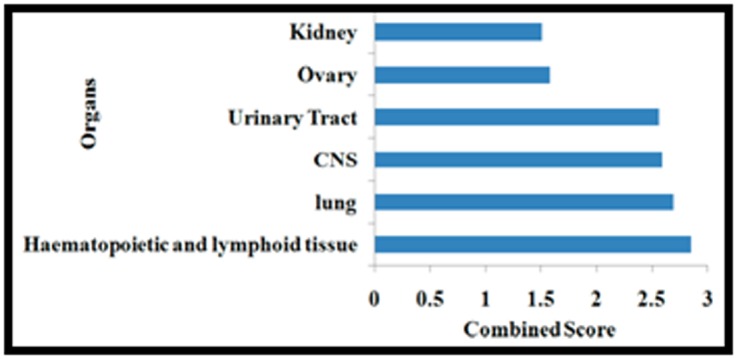
The bar graph shows Organs affected based on differential express genes from PCOS samples arrays with Enrichr.

These selected DEGs were then subjected to annotation in DAVID online tool. The functional classification of the DEGs was performed using the online gene ontology tool EnrichR, to check their relationship with biological processes, KEGG pathways and the most affected organs. The Figure 1, 2 and 3 show the overall graphical relationship and it is arranged according to the combined score. The combined score is computed by taking the log of the p-value from the Fisher exact test and multiplying by the z-score of the deviation from the expected rank. The further functional classification was performed using the online biological classification toolDAVID. The gene list was submitted with Homo sapiens as the background and was provided for enrichment calculation. Here we found the results to be overlapping with the EnrichR tool. The DAVID analysis revealed five genes (HHIP, WNT2, FGF10, NRG1, ROBO2) that were strongly associated with the most affected organs listed from the enrichR tool (Table 1). The functional annotation of gene classification, with their GO terms, p-value and that the study identified (Table 2) .The KEGG pathway associations of the obtained genes are reported (Table 3). Ten genes were enriched with several KEGG pathways, and ROBO2, CXCL2, EPHA5, EPHA6, ANGPT2, FGF10, GHF genes was found to be enriched with pathways influencing psychiatric disorders like Axon guidance, RAP1 Signaling pathways, RAS Signaling pathways etc.

## Discussion

Earlier studies have shown that PCOS is the most common endocrine abnormality in women of reproductive age and its prevalence is estimated to be 4-8% in Greece, Spain and in the USA [16,17]. The steady increase in the morbidity related to PCOS in the recent years indicates a need for further research on this disease [18]. Obesity and PCOS are linked as a result of a number of ways major ones include an altered lipid profile, excess adipose tissues – mass /cells; lymph tissues, increased ability to absorb fat, excess androgen hormone levels, use of psychiatric medications, altered hormonal profile and its link with brain, mainly hypothalamus, increased insulin resistance and its association with cAMP signaling, PI3K pathway and involvement of endometrial cells of obese PCOS women in other significant pathways, for example, Wnt, Shh, Etk etc.

Recently, Data analysis of Gene Expression has been used for disease biomarker discovery. It provides a way to develop better diagnostics, novel drug design strategies and biomarker identification and it helps in the improvement of clinical treatment efficacy. With the aim to discover novel biomarkers for polycystic ovary syndrome in obese women, 28 differentially expressed genes were discovered. All of these genes were found to be related with obesity and PCOS. The DEGs show a close reciprocity, prominently with endometrial and ovarian cancer. Uncontrolled growth in endometrial cells can either lead to a malignant tumor growth or endometrial hyperplasia, hyperplasty etc. Endometrial hyperplasia can lead to cancer also.

The pathway enrichment analysis of DEGs retrieved 9 genes and their associated pathways that were significantly enriched with GO terms and were associated with Axon guidance, Pathways in cancer, signaling pathways and other (Table 3). The psychiatric disorders are often influenced by axon guidance pathways [19, 20]. The psychiatric disorders include depression, anxiety, bipolar disorder, schizophrenia, posttraumatic stress disorder (PTSD), panic disorder, and substance use disorder. The number of obese PCOS women suffer from psychological issue is much larger than non-obese women with PCOS. This suggests that obese women with Polycystic Ovarian Syndrome (PCOS) are at a higher risk of developing or already facing mental health problem. Obesity and PCOS, however, can affect women mentally, independently also. Evidences clearly suggest that significant alterations in lipid profile, mostly caused by differential gene expression, and are a common factor in people with DBM II, obesity and mental illnesses, all of which are highly comorbid to PCOS. So we can state that obesity changes gene expression in women with PCOS.

In gene enrichment analysis, we found that the lung was the most affected organ (Figure 3) and 3 genes (WNT2, FGF10, HHIP) are involved in the normal lung development, induction, morphogenesis, and development of epithelium and branching of lungs. Along with lung, normal function of lymphoid tissue and hematopoietic tissue are also affected, or more specifically, decreased, at almost the same level on average relative to other organs. The other organs of which the fitness was decreased were CNS, Ovary and Urinary Tract. All the affected organs are shown in the Figure 3 below the length of the bar for a particular organ or tissue indicates the extent to which it is affected. Usually, organs with steep decrease in fitness are affected to a large extent too.

**Figure 5:**
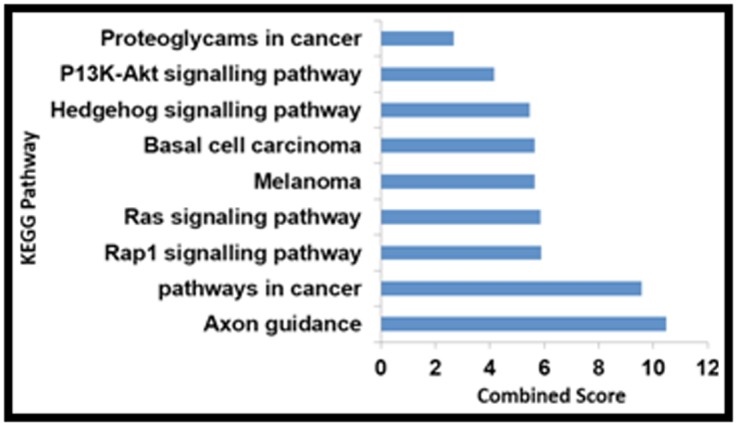
The bar graph shows Pathways associated with differential gene expression results using Enrichr.

The results of GO functional annotation and biological processes analysis retrieved nine genes that were significantly enriched with GO terms. Positive regulation of chemo taxis is one the biological processes which are intensely affected in obese women with PCOS (Table 2 and Figure 5). It can lead to abnormalities in autoimmune responses towards respiratory diseases like asthma, insulin-dependent DBM II etc. that are comorbid with PCOS. The inflammation response which is a characteristic of these diseases can halt since there will not be any more chemo attractants to draw immune cells towards the site of inflammation. This sudden halt may completely stop the inflammation or the inflammation will occur, but only partially (effects on phenotypes, genotypes and nearby cells, signaling molecules may change). Monocyte (immune related) chemotaxis is also affected but neither as much as positive chemotaxis nor to the extent as positive chemotaxis. Organ regeneration and regulation of synapse assembly, all of them are related to CNS, which was found to be a highly affected organ (Figure 3).

## Conclusion

Totally 28 genes were extracted from the study which are associated with obesity in PCOS. Polycystic ovary syndrome is a disorder of multiple hormones, glands, organs and systems in the body. Besides the ovaries, other organs and glands like the liver, thyroid, pancreas, hypothalamus, lungs, pituitary and adrenal glands are simultaneously affected. The cardiovascular system is definitely affected. The risk for future diseases is affected. The DEGs extracted from the study clearly shows the relation between obesity and PCOS and other risk factors involved with the disease. The Gene Enrichment Analysis suggests how Obesity alters several genes and associated pathways. This study can prove to be a fruitful foundation for the in vitro validation of the enriched genes, so that effective biomarkers could be achieved for treatment and prognosis of PCOS.

## Figures and Tables

**Table 1:** Gene names and Organs affected

Genes Names	Organs affected
HHIP, WNT2, FGF10	Lung
NRG1, ROBO2	Central Nervous System
ROBO2	Kidney

**Table 2:** GO Terms associated with genes and corresponding p value

Gene Names	GO Terms	P Value
CXCL2, ROBO2, ANGPPT2, FGF10, HGF, PLA2G7, ROBO2	Regulation of chemotaxis	8.61E-07
LRRTM4, GABRA3, PDLIM5	Regulation of Synapse Assembly	1.08E-05
ANGPT2, HGF, CXCLI2, FGF10	Organ Regeneration	4.04E-06
FGF10	Organ Induction	1.29E-05
CXCL12, PLA2G7	Positive regeneration of monocyte	3.49E-05
FGF10, CXCL12	Induction of positive chemotaxis	2.44E-04

**Table 3:** KEGG pathway associations of the genes

GENE Names	KEGG Pathways
ROBO2, CXCL2, EPHA5, EPHA6	Axon guidance
CXCL2, HHIP, WNT2, FGF10, HGF	Pathways in cancer
ANGPT2, FGF10, HGF	RAP1 Signaling Pathway, Ras Signaling Pathway
WNT2, FGF10, ANGPT2	Melanoma
HHIP, WNT2	Basal cell carcinoma
HHIP	Hedgehog signaling pathway
ANGPT2, FGF10	P13K-Akt signaling pathway
WNT2	Proteoglycans in cancer

**Figure 4:**
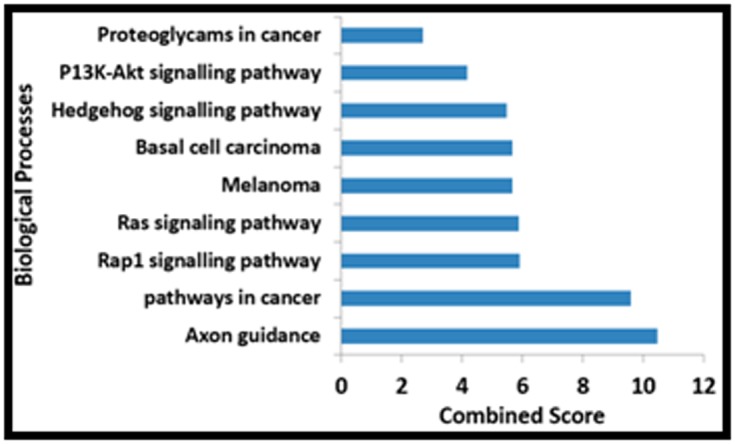
The bar graph shows differential expression of genes involve in biological process based on combined score from Enrichr.
